# Comparative Effects of Time-Restricted Eating and the Ketogenic Diet on QRISK3-Assessed Cardiovascular Risk in Individuals with Obesity: A Longitudinal Analysis of Metabolic, Anthropometric, and Lifestyle Factors

**DOI:** 10.3390/nu17121963

**Published:** 2025-06-09

**Authors:** Denisa Pescari, Monica Simina Mihuta, Andreea Bena, Dana Stoian

**Affiliations:** 1 Department of Doctoral Studies, “Victor Babeș” University of Medicine and Pharmacy, 300041 Timișoara, Romania; denisa.bostina@umft.ro; 2 Department of Advanced Ultrasound, DRD Medical Center, 300029 Timişoara, Romania; borlea.andreea@umft.ro (A.B.); stoian.dana@umft.ro (D.S.); 3 Center for Molecular Research in Nephrology and Vascular Disease, “Victor Babeș” University of Medicine and Pharmacy, 300041 Timișoara, Romania; 4 Department of Pediatrics, “Victor Babeș” University of Medicine and Pharmacy, 300041 Timișoara, Romania; 5 Discipline of Endocrinology, Second Department of Internal Medicine, “Victor Babeș” University of Medicine and Pharmacy, 300041 Timișoara, Romania

**Keywords:** obesity, cardiovascular risk, QRISK3, ketogenic diet, time-restricted eating

## Abstract

**Background/Objectives:** Obesity remains a major public health concern, and effective dietary strategies for reducing cardiovascular risk are still under investigation. This interventional non-randomized study aimed to evaluate the short-term effects of the modified ketogenic diet (KD) and time-restricted eating (TRE) on cardiovascular risk, as assessed by the QRISK3 score. **Methods**: Forty-nine adults with obesity were assigned to either the KD (n = 23) or TRE (n = 26), based on voluntary presentation to a nutrition clinic. Interventions were monitored by a certified clinical nutritionist over 12 weeks. Anthropometric parameters, metabolic markers, and QRISK3 scores were measured at baseline and post-intervention. **Results**: Both dietary approaches led to significant reductions in body weight and QRISK3 scores. However, the KD resulted in more pronounced improvements in the lipid profile, systolic blood pressure, and glycemic control. Interaction analysis suggested that older participants and those with a higher baseline risk benefited more from the KD. **Conclusions**: Over 12 weeks, both diets improved cardiovascular risk and metabolic markers in individuals with obesity. The modified diet ketogenic dietary pattern showed more pronounced short-term benefits in the lipid profile, blood pressure, and glycemic control. These results suggest its potential utility in clinical practice, though long-term effectiveness and safety remain to be established.

## 1. Introduction

Overweight and obesity are complex global health challenges associated with a broad range of medical, psychological, and social complications [1,2]. Obesity is a complex, heritable trait influenced by the interplay of genetics, epigenetics, metagenomics, and the environment [2]. According to recent epidemiological data, the prevalence of obesity has more than doubled since 1990, now affecting over 650 million adults worldwide [3].

Obesity is closely linked to cardiovascular diseases, type 2 diabetes, and increased overall mortality [3,4]. Managing individuals with excess weight presents numerous long-term challenges due to the multifactorial nature of obesity. Among contributing mechanisms, the interplay between the body’s energy conservation processes and an increased sensitivity to appetite-stimulating signals plays a significant role, making it particularly difficult to prevent weight regain after initial weight loss [4,5]. Long-term weight management is particularly challenging due to adaptive biological mechanisms that promote energy conservation and increase appetite after weight loss [5].

A range of interventions has been proposed to address these challenges, including dietary therapy, physical activity, behavioral modification, pharmacologic agents, and bariatric surgery [6]. Among these, dietary interventions remain the first-line strategy for weight reduction and cardiometabolic risk management [4,6].

However, weight reduction should not be regarded as the sole therapeutic objective. Instead, a comprehensive multidisciplinary approach aimed at targeting the cardiometabolic complications associated with obesity is imperative for the effective management of these patients. The concept of “diet” is broadly defined as the total energy and nutrient intake derived from the foods and beverages consumed regularly [7]. The modified ketogenic dietary pattern (KD), characterized by its low-carbohydrate and high-fat composition, has gained popularity as a weight loss intervention [8]. A key distinction between the ketogenic diet and other low-carbohydrate diets (LCDs) lies in its stricter carbohydrate restrictions, limiting daily intake to no more than 50 g or 10% of total macronutrient consumption [9]. The macronutrient composition of the ketogenic diet is typically distributed as 55–60% fats, 30–35% protein, and only 5–10% carbohydrates, which promotes the metabolic state of ketosis [10]. In this state, the body shifts from using carbohydrates to primarily metabolizing fats as its primary energy source, resulting in the production of ketone bodies [11]. The ketogenic diet offers a range of potential health benefits beyond weight loss, including reduced risk factors for type 2 diabetes, cardiovascular diseases, and certain cancers [12].

The ketogenic diet (KD) and time-restricted eating (TRE) have emerged as two promising dietary strategies. The KD is a high-fat, low-carbohydrate diet that promotes the metabolic state of ketosis, encouraging fat metabolism and ketone body production. It has shown benefits in weight loss, glycemic control, and lipid profile improvement [12]. TRE, in contrast, focuses on limiting the daily eating window—typically 4 to 10 h—without altering macronutrient content. It has been associated with improved insulin sensitivity, reduced blood pressure, and preservation of lean mass [13,14,15,16,17]. Nevertheless, recent findings have raised concerns about its long-term cardiovascular safety in certain populations, emphasizing the need for further study [18,19].

Cardiovascular risk can be assessed using the QRISK3 algorithm, a validated tool designed to estimate the 10-year probability of developing cardiovascular disease [20]. QRISK3 incorporates a comprehensive set of risk factors, including traditional variables (age, blood pressure, cholesterol levels) and additional ones such as BMI, ethnicity, socioeconomic deprivation, and comorbidities like chronic kidney disease, rheumatoid arthritis, and severe mental illness [21,22,23]. Although diet is not explicitly included in QRISK3, its influence is indirectly reflected through modifiable factors such as weight, blood pressure, and lipid levels [24].

This interventional non-randomized study aims to compare the effects of a 12-week modified ketogenic dietary pattern and time-restricted eating intervention on QRISK3 scores in individuals with obesity. By analyzing the dynamics of cardiovascular risk alongside changes in BMI, blood pressure, lipid profile, and body composition, the study seeks to evaluate the potential of these two distinct dietary strategies in reducing long-term cardiovascular risk and improving overall metabolic health.

## 2. Materials and Methods

This interventional non-randomized study was conducted from July 2022 to April 2024, within the endocrinology and nutrition unit. A total of 49 participants with overweight or obesity were enrolled, with 26 assigned to the TRE group and 23 to the KD group. The mean age was 35.23 ± 11.16 years. All participants voluntarily enrolled in the study after seeking nutritional intervention at our center with the goal of lifestyle modification and potential weight optimization. The study aimed to implement evidence-based lifestyle modifications and tailored dietary interventions to support weight reduction and improve metabolic and cardiovascular health. Clinical and nutritional evaluations, including QRISK3 score calculations, were conducted at baseline and after 12 weeks by a certified clinical nutritionist. Notably, no adverse events were reported in either group during the study. While this information was not initially highlighted, it supports the short-term safety and tolerability of both dietary interventions in a supervised setting.

Informed consent was obtained from all participants prior to their inclusion in the study. The research was conducted in compliance with the ethical principles outlined in the Helsinki Declaration and received formal approval from the Scientific Research Ethics Committee (CECS) of the “Victor Babeș” University of Medicine and Pharmacy Timișoara (Approval No. 69/3 October 2022).

### 2.1. Patient Inclusion and Exclusion Criteria

Inclusion Criteria:

◦ Adults aged 25 years or older with a BMI > 25 kg/m^2^ seeking nutritional counseling for weight loss.
◦ Individuals from both urban and rural environments, representing diverse educational backgrounds.
◦ Women at different menopausal stages (both menopausal and non-menopausal).
◦ Participants who
  - Provided signed informed consent.
  - Completed all scheduled evaluations and demonstrated adherence to the prescribed dietary program throughout the study period were included in the final analysis.

Exclusion Criteria:

◦ Physiological Conditions:
  - Pregnant or breastfeeding women.
◦ Medical and Pharmacological Conditions:
  - Use of dietary supplements or anti-obesity medications [25].
  - Patients with diabetes mellitus treated with:
  - Oral hypoglycemic agents associated with a risk of hypoglycemia (e.g., sulfonylureas) [26].
  - Insulin therapy.
  - History of dietary therapy in the past 12 months.
  - History of bariatric surgery.
  - Acute pancreatitis, renal or liver diseases (including chronic kidney disease and liver failure).
  - Porphyria diagnosis.
◦ Obesity Due to Specific Etiologies:
  - Genetic conditions (e.g., Prader–Willi syndrome).
  - Iatrogenic causes (e.g., insulin therapy, corticosteroid therapy, antipsychotics).
  - Endocrinological disorders (e.g., Cushing’s syndrome, hypothyroidism, hypogonadism) [27].
◦ Lifestyle and Compliance Issues:

Subjects who consumed alcohol above the recommended moderate intake levels (men consuming two or more drinks per day and women consuming one or more drinks per day). One standard drink was defined based on established guidelines as follows [28]:

- 12 ounces (355 mL) of beer (5% alcohol by volume).
- 8 ounces (237 mL) of malt liquor (7% alcohol by volume).
- 5 ounces (148 mL) of wine (12% alcohol by volume).
- 1.5 ounces (44 mL) of liquor or distilled spirits (40% alcohol by volume, also known as 80-proof liquor).

Failure to

- Adhere to the prescribed dietary program.
- Attend scheduled medical visits.

### 2.2. Comprehensive Clinical Evaluation

Prior to any procedures, all participants received detailed information about the study’s objectives, methodology, and required clinical and paraclinical evaluations.

During the initial consultation:

- A comprehensive anamnesis was conducted to collect data on demographic characteristics, medical history, behavioral factors, and laboratory results from the previous six months.
- The QRISK3 score was calculated, interpreted, and explained, including the contributing variables, the absolute score, and the relative risk score.
  The primary non-invasive technique used was bioelectrical impedance analysis (BIA) for precise segmental body composition assessment. Based on these parameters, participants were stratified into subgroups for further analysis.
  ⇒ Personal medical history: A detailed anamnesis assessed comorbidities relevant to QRISK3, including hypertension, diabetes, kidney disease, and cardiovascular risk factors. Family history of early angina or myocardial infarction was also recorded. Recent biological data, including glucose, lipid profile, uric acid, HbA1c, TSH, FT4, HOMA-IR, and 25-OH vitamin D, were collected for participant stratification. Menopausal status was documented for its impact on metabolic and cardiovascular risk.
  ⇒ Factors related to behavior and lifestyle evaluated were as follows:

◦ Physical activity level: Participants needed at least 150 min of moderate to vigorous exercise weekly or 30 min daily to avoid being classified as sedentary.
◦ Sleep duration: Less than 7 h of sleep per night was considered sleep deprivation, following established guidelines [29].
◦ Alcohol consumption: Participants self-reported alcohol intake, with one unit defined as 10 mL of pure ethanol. Those consuming over two units per day were classified as having chronic alcohol use, while abstainers were categorized as non-drinkers [28,30].
◦ Smoking status: Participants were categorized as non-smokers, ex-smokers, or smokers (light: <10 cigarettes/day, moderate: 10–19/day, heavy: ≥20/day) for QRISK3 assessment.
  ⇒ Nutritional status was evaluated using BMI, calculated as weight (kg)/height^2^ (m^2^) [31,32]. All measurements were conducted by a trained physician to ensure precision and reliability. To minimize variability, assessments were standardized by performing them at the same time of day, with participants advised to avoid intense physical activity and hydration fluctuations prior to evaluation.
◦ Height Measurement: Height was assessed using a calibrated wall-mounted stadiometer, with participants standing upright, barefoot, and aligned for accuracy.
◦ Body Weight Measurement: Weight was recorded using a certified mechanical scale (max. 180 kg), with participants standing upright, wearing minimal clothing, and without footwear.
◦ Circumference Measurements: Waist circumference was measured at the midpoint between the last palpable rib and the iliac crest, while hip circumference was taken at the widest buttock region, using a non-elastic, calibrated tape parallel to the floor.
◦ Waist-to-Hip Ratio (WHR): WHR was calculated as waist circumference (cm) divided by hip circumference (cm), ensuring standardized positioning and minimizing measurement error.

### 2.3. QRISK3 Score Calculation and Cardiovascular Risk Assessment

The QRISK3 algorithm has been validated in large cohort studies and is widely used in clinical practice to guide decisions regarding interventions for CVD prevention [20,33]. The QRISK3 score was calculated at baseline and after 12 weeks to assess cardiovascular risk dynamics. Using the validated QRISK3 algorithm, which integrates demographic, clinical, and biochemical parameters, the score estimated the 10-year CVD risk. Calculations were performed with the official University of Nottingham online tool to ensure accuracy. Data were carefully entered, and results interpreted based on clinical thresholds. The variables included in the QRISK3 calculation were as follows:

- Age (years).
- Sex (male/female).
- Ethnicity.
- Body mass index (BMI) (kg/m^2^).
- Systolic blood pressure (mmHg).
- Total cholesterol to high-density lipoprotein cholesterol (TC/HDL) ratio.
- Smoking status (non-smoker, ex-smoker, light smoker < 10 cigarettes/day, moderate smoker 10–19 cigarettes/day, heavy smoker ≥ 20 cigarettes/day).
- Diagnosis of hypertension.
- Diagnosis of type 2 diabetes mellitus.
- Diagnosis of chronic kidney disease (stages 3, 4, or 5).
- Diagnosis of rheumatoid arthritis.
- Diagnosis of systemic lupus erythematosus.
- History of atrial fibrillation.
- History of migraine.
- Diagnosis of severe mental illness (e.g., schizophrenia, bipolar disorder, major depression).
- Use of atypical antipsychotic medication.
- Regular corticosteroid therapy.
- Presence of erectile dysfunction (in male participants).
- Family history of premature cardiovascular disease (angina or myocardial infarction before the age of 60 in a first-degree relative) [20,34].

By incorporating a comprehensive range of risk factors, QRISK3 provides a nuanced assessment of an individual’s cardiovascular risk, facilitating personalized management strategies [33].

### 2.4. Clinical Weight Management Intervention

Following the initial clinical and nutritional evaluation, each participant was assigned a structured dietary intervention by a certified clinical nutritionist, to be maintained consistently over a 12-week period. Individualized caloric and macronutrient distributions were determined based on a comprehensive assessment of baseline nutritional status, ensuring adherence to the specific dietary framework designated for each participant. The dietary protocols were strictly implemented throughout the 12-week intervention, with meals organized into three main meals and two snacks per day. Food selection followed national regulatory guidelines, requiring participants to carefully analyze nutritional labels to ensure compliance [35]. In addition to personalized dietary plans, general nutritional recommendations were provided to support overall metabolic health. These guidelines emphasized a minimum daily intake of 2500 mL of non-caloric fluids, the consumption of unsweetened coffee without milk, and the exclusion of any food items not included in the assigned dietary regimen for the duration of the study. To ensure adherence and compliance with the intervention, daily dietary intake was monitored through an online tracking platform. Additionally, participants attended weekly nutritional follow-up visits at the medical center, where adherence and dietary adjustments were assessed. BIA was performed at two key time points, baseline and at the conclusion of the program, to evaluate changes in body composition and the impact of the prescribed dietary interventions. The dietary interventions were determined through a collaborative decision-making process involving the physician, clinical nutritionist, and participant, following a comprehensive initial nutritional assessment. The interventions were categorized into two distinct dietary approaches: isocaloric diets with a predefined macronutrient distribution, represented by the KD, and isocaloric diets without a specific macronutrient distribution, represented by TRE. The final caloric intake for each participant was individually calculated based on their BMR, which was measured via BIA at baseline. This value was then adjusted by multiplying it with the participant’s physical activity level (PAL), using the midpoint value corresponding to a moderately active lifestyle, ensuring an accurate estimation of total daily energy expenditure [36,37].

The KD was structured to ensure strict carbohydrate restriction, with total daily carbohydrate intake maintained at ≤10% of total energy consumption, not exceeding 50 g per day [11]. In this dietary protocol, carbohydrates were predominantly replaced by healthy fats [38] and complete, bioavailable protein sources [39], while ensuring that the overall caloric intake remained isocaloric. A modified ketogenic dietary pattern (KD) was implemented. In this approach, fat intake accounted for approximately 60% of total daily energy intake, protein contributed around 30%, and carbohydrate intake was limited to a maximum of 50 g per day. Macronutrient distribution was adjusted to meet individual energy requirements [10]. TRE protocol was structured around a defined eating window ranging from 4 to 10 h, varying according to individual adherence capabilities and metabolic response [13]. Unlike the KD intervention, TRE did not impose explicit macronutrient distribution constraints. Instead, participants were allowed ad libitum food consumption within their designated eating window while maintaining compliance with the fasting protocol during the remaining 14 to 20 h. Throughout the fasting phase, participants were instructed to maintain adequate hydration levels and were permitted to consume non-caloric fluids, such as water, herbal teas, and black coffee. To ensure adherence and dietary compliance, all meals consumed during the intervention were monitored daily by a certified nutritionist via an online tracking platform.

### 2.5. Statistical Analysis

A comprehensive statistical approach was employed to assess the impact of TRE and the KD on QRISK scores, as well as related anthropometric and metabolic outcomes. The analysis aimed to evaluate baseline differences between the dietary groups, assess changes over time, explore correlations between QRISK changes and other variables, and investigate interaction effects of key predictors with diet type on QRISK reduction.

As this was a real-life, interventional non-randomized study based on a voluntary cohort presenting to our center, no a priori sample size calculation was performed. All statistical analyses were conducted on the available sample, using appropriate methods to ensure the validity of the results.

Continuous variables were summarized using medians and interquartile ranges (IQRs), while categorical variables were summarized using counts and percentages. The Mann–Whitney U test was employed to compare continuous variables between the IF and KD groups, as the non-normal distribution of most variables was confirmed using the Shapiro–Wilk test. Rank-biserial correlation ($\hat{r}$) was calculated to estimate the effect sizes. Categorical variables were compared between groups using the Pearson chi-square test. To evaluate longitudinal changes in variables measured at both the start and end of the study, the Wilcoxon signed-rank test was used, stratified by diet type. This approach provided insight into within-group changes over time. Spearman’s rank correlation analysis was conducted to examine the relationships between the change in QRISK (QRISK end–QRISK start) and other numerical variables within each diet group, allowing for the identification of key factors associated with cardiovascular risk reduction.

A multivariate linear regression model was constructed to evaluate predictors of final QRISK scores. Predictors were selected using the backwards elimination method and were included to determine their independent contributions to cardiovascular risk at the end of the study. Estimates, confidence intervals (CI), and *p*-values were reported for all predictors.

The interaction effects between key predictors and diet type on the change in QRISK were modeled using linear regression. Interaction terms were included to assess whether the relationship between variables such as age, BMI, systolic blood pressure (SBP), fasting glucose, HbA1c, WHR, and menopausal status differed based on diet type. Adjusted R^2^ values and *p*-values were reported to quantify the explanatory power and statistical significance of these models.

All statistical analyses were performed at a significance level of *p* < 0.05. Visualizations, including interaction plots and scatterplots, were used to illustrate key findings and highlight significant relationships between predictors and outcomes. Data analysis was conducted using R (version 4.3.0) and RStudio (version 2023.06.0+421) to ensure the robustness, reproducibility, and clarity of the results.

## 3. Results

This study followed an interventional non-randomized design, and all participants included in the final analysis strictly met the predefined inclusion and exclusion criteria prior to enrollment. No additional post hoc exclusions were made based on dietary adherence, in order to preserve ecological validity. While the study followed a non-randomized interventional design, the absence of protocol deviations and the real-world setting in which dietary interventions were self-prepared by participants at home reflect the conditions encountered in routine clinical practice.

### 3.1. Baseline Characteristics of Participants Across Dietary Groups

The baseline characteristics of the participants were compared between the TRE and KD groups to ensure that any observed differences in outcomes were not confounded by pre-existing disparities (Table 1). Mann–Whitney U tests were employed to assess statistical differences between the two dietary interventions. The age distribution was similar between the two groups, with no significant difference observed (*p* = 0.790). At baseline, participants in the KD group had a significantly higher BMI (*p* = 0.020), waist circumference (*p* = 0.040), and waist-to-hip ratio (*p* = 0.040) compared to those in the IF group, suggesting potential differences in body composition between the groups.

Lipid profile parameters, including TC, HDL-C, TC/HDL-C ratio, non-HDL cholesterol, LDL-C, and triglycerides, did not show significant baseline differences between the dietary groups (all *p*-values > 0.05), indicating a comparable cardiovascular risk profile at the start of the study. Similarly, SBP was not significantly different (*p* = 0.790), further supporting the homogeneity of cardiovascular risk factors between groups. Importantly, the primary risk assessment tool in this study, QRISK, showed no significant difference between the IF and KD groups at baseline (*p* = 0.700). QRISK scores were calculated at baseline and after 12 weeks using the validated QRISK online calculator, without any modifications to its components. All risk factors contributing to QRISK were collected according to standard clinical and biochemical assessments at both time points, ensuring consistency. The application of QRISK in this study follows established protocols used in previous dietary intervention trials assessing cardiovascular risk dynamics.

Markers of glucose metabolism, including fasting glucose (*p* = 0.480), HbA1c (*p* = 0.620), and HOMA-IR (*p* = 0.140), did not significantly differ at baseline, indicating that insulin sensitivity and glycemic control were not substantially different prior to dietary intervention. Similarly, serum creatinine and uric acid levels were comparable (*p* = 0.410 and *p* = 0.160, respectively).

Lastly, vitamin D levels, while slightly higher in the TRE group, did not reach statistical significance (*p* = 0.140), suggesting that baseline vitamin D status was relatively similar between the dietary groups.

The baseline categorical characteristics of the study participants were analyzed using the Pearson chi-square test to determine whether there were significant differences between the TRE and KD groups. The results, summarized in Table 2, indicate that most categorical variables were evenly distributed between the two dietary interventions, suggesting comparable baseline conditions. The gender distribution was similar in both groups, with females representing the majority of participants in both the TRE (65.38%) and KD (69.57%) groups (*p* = 0.760). Similarly, the proportion of smokers did not differ significantly between the two groups (*p* = 0.790), nor did the proportion of sedentary individuals (*p* = 0.790) or those experiencing sleep deficits (*p* = 0.420). The prevalence of menopause was also comparable (*p* = 0.670), suggesting that hormonal differences were unlikely to confound the results. Regarding cardiovascular risk factors, a family history of cardiovascular disease (FMH CV) was more prevalent in the TRE group (53.85%) than in the KD group (30.43%), though this difference did not reach statistical significance (*p* = 0.100). The presence of lupus erythematosus (LES) and erectile dysfunction (ED) was not significantly different between groups (*p* = 0.520 and *p* = 0.290, respectively). These comorbidities were assessed due to their known associations with systemic inflammation and cardiovascular risk. The presence of atrial fibrillation (AF) was rare in both groups, with only one case in the IF group and none in the KD group (*p* = 0.340).

### 3.2. Longitudinal and Comparative Analysis of Health Markers

We first assessed within-group changes using the Wilcoxon signed-rank test for each variable measured at baseline and post-intervention, stratified by dietary group. To compare the magnitude of change between groups, Mann–Whitney U tests were applied, along with effect size estimates. This combined approach allows for a more accurate evaluation of the dietary interventions’ effectiveness.

Percentage changes were calculated for each participant by comparing post-intervention to baseline values using the formula: Percentage Change = (Baseline − Final)/Baseline × 100. This approach standardizes improvements across groups despite baseline differences. The median percentage change and interquartile range (IQR) were reported for each variable, and statistical comparisons were conducted using the Wilcoxon signed-rank test (within-group changes) and Mann–Whitney U test (between-group comparisons). The results are presented in Table 3.

Both dietary interventions led to significant relative reductions in BMI, with the KD group achieving a greater median percentage change (12.71%, IQR: 9.84, 16.66) compared to the TRE group (4.06%, IQR: 2.41, 6.49; *p* < 0.001 for between-group comparison). Waist circumference (WC) showed a similar pattern, with the KD group experiencing a larger median percentage reduction (11.43%, IQR: 8.75, 13.45) than the TRE group (1.73%, IQR: 0.91, 3.15; *p* < 0.001). For waist-to-hip ratio (WHR), the KD group again demonstrated a greater improvement (11.22%, IQR: 6.42, 19.21) relative to the TRE group (2.33%, IQR: 0.28, 5.65; *p* < 0.001). These findings indicate that while both diets positively impacted adiposity, the modified ketogenic dietary pattern was associated with more pronounced body composition improvements.

In terms of lipid profile, total cholesterol (TC) decreased significantly in both groups, with a greater relative reduction in the KD group (18.03%, IQR: 14.89, 19.88) compared to the TRE group (2.04%, IQR: 0.23, 3.99; *p* < 0.001). HDL-C improved markedly in the KD group (12.73%, IQR: 8.33, 20.20), while the TRE group experienced a slight decrease (–2.07%, IQR: –5.02, 1.62; *p* < 0.001). The TC/HDL-C ratio also decreased more substantially in the KD group (25.74%, IQR: 23.13, 30.50) compared to the TRE group (–0.76%, IQR: –2.78, 4.67; *p* < 0.001), reflecting a more favorable shift in cardiovascular risk markers under the KD.

Systolic blood pressure (SBP) declined significantly in both groups, with the KD group showing a larger relative reduction (8.97%, IQR: 3.41, 12.50) than the TRE group (1.42%, IQR: 0, 2.94; *p* < 0.001). Cardiovascular risk, as assessed by QRISK and relative risk scores, improved more prominently in the KD group: QRISK decreased by 38.98% (IQR: 29.79, 45.38) vs. 2.16% (IQR: 0.74, 8.03) in the TRE group, and relative risk decreased by 26.76% (IQR: 18.74, 44.78) vs. 1.61% (IQR: 0.58, 5.44), both with *p* < 0.001.

Lastly, glycemic control improved in both groups, with a modest reduction in HbA1c in the TRE group (1.58%, IQR: 0, 1.95) and a substantially greater improvement in the KD group (8.33%, IQR: 2.72, 13.60; *p* < 0.001). These results, summarized in Table 3, suggest that the KD was more effective in improving anthropometric, metabolic, and cardiovascular risk markers compared to time-restricted eating.

Given the significant between-group differences in percentage change for key variables, a post hoc power analysis was performed to evaluate the robustness of these findings. The analysis focused on Mann–Whitney U tests comparing the relative (% change) values between the TRE and KD groups. The results indicated adequate statistical power for detecting differences in BMI (power = 0.804), WC (0.911), and TC (0.901) changes. Power was somewhat lower for WHR (0.570), SBP (0.660), and HbA1c (0.584), suggesting that larger sample sizes may be needed to confirm these findings with greater certainty. Full results are presented in Table 4.

**Figure 1:**
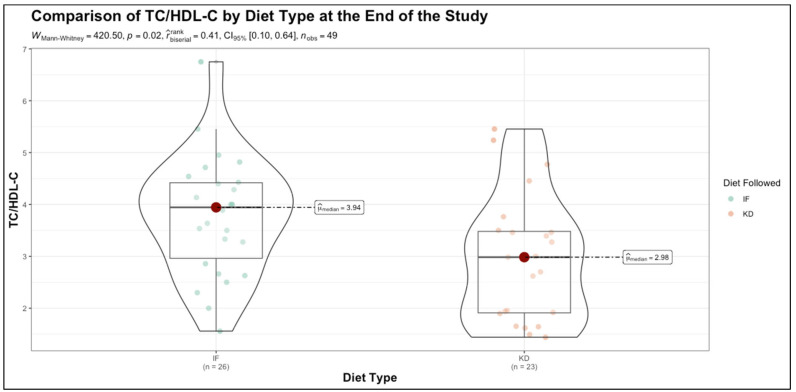
Distribution of TC/HDL-C ratio by diet type at the end of the study. Abbreviations: TC—total cholesterol (mg/dL); HDL-C—high-density lipoprotein cholesterol (mg/dL); TC/HDL-C—total cholesterol to high-density lipoprotein ratio; IF—intermittent fasting; KD—ketogenic diet.

To further visualize one of the key between-group differences, Figure 1 illustrates the distribution of TC/HDL-C ratios at the end of the intervention. As shown, participants in the KD group had a significantly lower TC/HDL-C ratio compared to the TRE group (*p* = 0.020), consistent with the broader findings of a more favorable lipid profile under the KD.

## 4. Discussion

Dietary habits, lifestyle factors, psychological influences, and genetics collectively shape nutritional status, regulating adipose and lean tissue distribution. Understanding these interactions is crucial for optimizing dietary interventions for weight management and metabolic health. As a key risk factor for cardiovascular diseases, obesity triggers metabolic, inflammatory, and hemodynamic disturbances that elevate morbidity and mortality [40]. Additionally, obesity is strongly associated with hypertension, driven by heightened sympathetic activity, renin–angiotensin–aldosterone system activation, and impaired natriuresis, all contributing to elevated blood pressure and cardiac strain [41].

Given the strong link between obesity and cardiovascular disease, early risk stratification is crucial for timely intervention. The QRISK3 score is a well-validated cardiovascular risk prediction tool that provides an advanced assessment of an individual’s likelihood of developing CVD over the next ten years [20,42]. The inclusion of obesity as a weighted risk factor acknowledges its independent contribution to atherosclerosis, hypertension, and cardiac dysfunction, making QRISK3 a superior tool for identifying high-risk individuals who may otherwise be overlooked [43]. One of the key advantages of QRISK3 in obese populations is its ability to refine risk prediction beyond traditional models such as Framingham or QRISK2, which primarily focused on conventional risk factors like hypertension, cholesterol levels, smoking, and diabetes [20,44]. Obesity, particularly central adiposity, has been shown to accelerate cardiovascular aging by inducing metabolic disturbances, systemic inflammation, and vascular dysfunction, making its inclusion in risk assessment models essential [45]. Additionally, QRISK3 accounts for conditions that frequently co-exist with obesity, such as atrial fibrillation and migraines, both of which further elevate cardiovascular risk [44]. The clinical utility of QRISK3 is particularly relevant for guiding preventative interventions in obese individuals. By providing a more accurate estimation of absolute cardiovascular risk, clinicians can better tailor treatment strategies, including lifestyle modifications, lipid-lowering therapy, antihypertensives, and, in some cases, metabolic or bariatric surgery [46,47]. Furthermore, QRISK3 helps to stratify patients who may require more aggressive monitoring or early initiation of pharmacological interventions, such as GLP-1 receptor agonists, which have demonstrated cardioprotective benefits in individuals with obesity-related metabolic syndrome [48]. Given the increasing prevalence of obesity worldwide, integrating QRISK3 into routine clinical practice allows for a more personalized and precise approach to cardiovascular risk assessment. Its ability to capture the multifaceted contributions of obesity to CVD risk ensures that high-risk individuals receive appropriate, early interventions, ultimately reducing cardiovascular morbidity and mortality [49].

Although QRISK3 is primarily validated for long-term cardiovascular risk estimation, its application in this study was justified by its sensitivity to clinically relevant, modifiable parameters such as BMI, HbA1c, systolic blood pressure, and lipid profile, all of which are known to exhibit measurable changes over relatively short intervention periods. The rationale was not to predict immediate cardiovascular events, but to assess whether early metabolic improvements resulting from dietary intervention could influence projected cardiovascular risk trajectories. Moreover, QRISK3 incorporates a broader range of demographic and clinical variables, allowing for a more comprehensive and individualized assessment of risk, particularly in populations with obesity and associated metabolic disturbances. Its use in this context enhances the translational relevance of our findings by linking short-term physiological changes to longer-term risk indicators widely used in preventive cardiometabolic care. Therefore, the widespread adoption of QRISK3 in obesity management is essential for improving cardiovascular outcomes and reducing healthcare burdens associated with obesity-related complications [50].

Dietary therapy remains the first-line approach for obesity management before pharmacological or surgical options [51]. While multiple factors influence weight loss, meal timing, macronutrient composition, and caloric intake play key roles. Research continues to explore optimal dietary strategies, emphasizing energy deficit as the primary driver of weight reduction [52,53,54].

TRE and the KD influence metabolism and cardiovascular health through distinct mechanisms. TRE improves insulin sensitivity, reduces inflammation, and lowers blood pressure without altering macronutrient intake. The KD, while promoting weight loss and lowering triglycerides, has mixed cardiovascular effects, particularly concerning increased LDL-C levels [55]. Our study found both TRE and the KD effective for weight management, but notable differences emerged in cardiovascular and lipid parameters. The KD group showed increased HDL-C and a lower TC/HDL-C ratio, suggesting a favorable impact on lipid metabolism. Additionally, the significant reduction in relative cardiovascular risk in the KD group highlights its potential role in long-term cardiovascular health. Despite these promising findings, the lack of significant differences in glycemic control parameters suggests that both dietary strategies exert comparable effects on glucose metabolism. The trend toward improved QRISK scores and SBP reduction in the KD group warrants further investigation to confirm its potential long-term benefits.

The differential effects of the KD and TRE may be attributed to their distinct metabolic mechanisms. The KD induces ketosis through carbohydrate restriction, enhancing lipid oxidation and improving markers such as HDL-C and the TC/HDL-C ratio. In contrast, TRE primarily influences circadian and hormonal regulation without altering macronutrient intake, resulting in more modest short-term changes. These physiological differences likely explain the superior short-term cardiometabolic improvements observed in the KD group.

Given the study’s limitations, including sample size and intervention duration, future research should explore the mechanistic underpinnings and clinical implications of these dietary interventions in larger, more diverse populations. Previous studies have demonstrated the impact of both ketogenic diets and time-restricted eating on cardiometabolic parameters.

Although some between-group differences approached but did not reach conventional levels of statistical significance (e.g., systolic blood pressure, *p* = 0.10; HbA1c, *p* = 0.13), these trends may still hold clinical relevance. In preventive cardiology and metabolic care, even modest short-term improvements in blood pressure, glycemic control, or estimated cardiovascular risk scores can have meaningful long-term benefits if sustained. Additionally, the observed changes in these parameters were directionally consistent with the expected physiological responses to dietary interventions. Effect size estimates and post hoc power analyses further support the clinical value of the findings, suggesting that the magnitude of change, particularly in the KD group, may be relevant even in the absence of strong statistical signals due to limited sample size. Therefore, these results should be interpreted as potentially meaningful, especially when considered in the context of real-world, individualized dietary therapy.

Conversely, while TRE has been associated with modest weight loss and reductions in blood pressure and triglyceride levels, recent studies have raised concerns about its long-term cardiovascular safety. An analysis of over 20,000 adults found that an 8 h TRE schedule was linked to a 91% higher risk of cardiovascular mortality compared to a 12–16 h eating window [56]. The present study found no significant differences in glycemic control between the KD and TRE, suggesting similar effects on glucose metabolism. This aligns with previous research showing both diets can improve glycemic parameters, though their long-term impact may vary [55].

While both TRE and the KD support weight management, the KD appears to provide additional benefits in lipid profile improvement and cardiovascular risk reduction. However, the long-term effects of TRE require further study. Future research should explore the underlying mechanisms and assess the clinical relevance of these dietary strategies in diverse populations. Our study’s interaction analysis between baseline BMI and dietary intervention on changes in QRISK scores indicates that the KD may offer enhanced cardiovascular benefits for individuals with elevated BMI. In contrast, while TRE has been associated with modest weight loss and improvements in metabolic health, its long-term cardiovascular benefits remain uncertain. Furthermore, our analysis of the interaction between WHR and diet type on QRISK changes revealed that a higher baseline WHR was associated with more substantial reductions in QRISK for participants following the KD compared to those on TRE. This suggests that the KD may be particularly effective in mitigating cardiovascular risk factors related to central adiposity.

While this study demonstrated favorable short-term outcomes for both the modified ketogenic dietary pattern and time-restricted eating, particularly with regard to weight loss and cardiometabolic risk reduction, long-term adherence and safety remain key challenges for clinical implementation. The KD, although effective in producing rapid metabolic improvements, may be associated with lower long-term adherence due to its restrictive nature and potential concerns related to lipid profiles and nutrient adequacy. Similarly, although time-restricted eating is generally perceived as more sustainable, data on its prolonged effects and adherence rates remain limited. Future studies with extended follow-up periods are needed to evaluate the durability, acceptability, and safety of these dietary strategies over time, particularly in diverse clinical populations.

Despite its valuable findings, our study has several limitations. First, the sample size was relatively small, which may limit the generalizability of the results to broader populations. Larger-scale studies with diverse cohorts are necessary to validate our findings. Second, the duration of the intervention was limited, and long-term adherence to dietary interventions and their sustained effects on cardiovascular risk were not assessed. Future studies should include extended follow-up periods to evaluate the durability of these effects. Self-reported dietary intake and compliance could introduce measurement biases. Lastly, while the study accounted for key metabolic and cardiovascular markers, other confounding variables, including genetic predisposition were not extensively analyzed. Addressing these factors in future research could provide a more comprehensive understanding of dietary interventions on cardiovascular health. Another key limitation of this study is participant selection, as inclusion was based on voluntary presentation to our clinic for nutritional assessment. Selection was random, based on the order of patient visits, but without a predefined randomized allocation, which may introduce selection bias. Additionally, the allocation can be considered a form of mutual agreement, as participants were informed about the study design and voluntarily continued in their assigned group. Regarding attrition, only patients who fully adhered to the prescribed dietary intervention (TRE or the KD) were included in the final analysis. Future studies should consider randomized allocation and longer follow-up to better assess the long-term effects of these dietary interventions. Moreover, the prevalence of RA and LES among study participants was slightly higher than expected in the general population. However, this was coincidental rather than a result of specific inclusion criteria. Given that neither condition was a primary focus of the study and no significant differences in outcomes were observed between participants with or without these conditions, their presence is unlikely to have influenced the results. Additionally, while some individuals were receiving corticosteroids or immunosuppressive therapy, no significant interaction between these treatments and study outcomes was identified. An unexpected finding in this study was the higher prevalence of migraine patients in the TRE group. This was not due to selection bias, as allocation was not based on migraine status, but rather an incidental imbalance in participant distribution. While time-restricted eating has been investigated for its potential effects on migraine frequency and severity, this study did not assess headache-related outcomes. This difference in distribution is unlikely to have impacted the primary findings related to QRISK3 score changes.

An important limitation of this study is the absence of biochemical confirmation of ketosis. Although the dietary composition was intended to induce a ketogenic state, no measurements of ketone bodies were performed to verify whether participants actually achieved and maintained nutritional ketosis. As such, the classification of the intervention as a “ketogenic diet” remains theoretical and cannot be definitively substantiated without biochemical evidence.

Another important limitation of this study is the difference in baseline anthropometric measurements between the two groups, with the KD group presenting a higher BMI, waist circumference, and waist-to-hip ratio. This imbalance is not the result of random allocation but rather reflects the real-life, voluntary nature of patient selection and counseling in our clinical setting. Dietary interventions were recommended based on a comprehensive assessment of metabolic status, body composition, and obesity severity and were chosen in mutual agreement with each patient. While this approach mirrors routine clinical practice, it may introduce bias in outcome interpretation and should be considered when evaluating the results.

Therefore, the main limitations of the present research are the small sample size, which may impact the generalizability of the findings, and the short follow-up period, which does not allow for the assessment of long-term adherence and sustained effects on cardiovascular risk.

## 5. Conclusions

This study suggests that both the modified ketogenic dietary pattern and time-restricted eating may offer promising short-term benefits in improving body composition and cardiovascular risk markers in individuals with obesity. While both interventions led to reductions in weight and waist circumference, the ketogenic diet appeared to produce more noticeable changes in lipid profiles, glycemic control, and blood pressure. The reductions observed in QRISK3 scores indicate potential short-term cardiovascular improvements, particularly among individuals with metabolic dysfunction and postmenopausal women. These effects were more evident in participants with higher baseline BMI, fasting glucose, and cardiovascular risk.

However, given the limited sample size and short duration of the study, these findings should be interpreted with caution. Rather than drawing definitive conclusions about the superiority of one strategy over another, our results should be viewed as preliminary evidence supporting the need for larger, long-term studies to explore the feasibility, adherence, metabolic impact, and clinical relevance of these dietary approaches over time.

## Figures and Tables

**Table 1:** Baseline numerical characteristics of participants in the time-restricted eating and ketogenic diet groups.

Variable	TRE N = 26	KD N = 23	*p*-Value
Age	37.50 (28.25–48.00)	36.00 (29.50–42.50)	0.790
BMI	30.3 (27.4–33.2)	33.20 (31.9–37.8)	0.020
WC	100.50 (91.00–110.75)	110.00 (97.50–122.50)	0.040
WHR	0.90 (0.88–1.07)	0.98 (0.92–1.17)	0.040
TC	187 (167–219)	200 (145–242)	0.860
HDL-C	54 (45–60)	48 (46–60)	0.600
TC/HDL-C	3.85 (3.01–4.30)	3.91 (2.52–4.98)	0.860
SBP	126 (112–143)	122 (115–145)	0.790
QRISK	11.75 (6.58–30.12)	11.80 (7.15–26.15)	0.700
Relative Risk	16.70 (8.27–33.83)	13.00 (10.10–22.20)	0.620
Non-HDL-C	139 (104–170)	155 (85–187)	0.640
LDL-C	124 (94–143)	131 (85–163)	0.680
Triglycerides	100 (86–164)	120 (87–173)	0.600
Uric Acid	5.75 (4.67–6.60)	5.20 (4.05–5.95)	0.160
Serum Creatinine	0.67 (0.65–0.71)	0.64 (0.58–0.72)	0.410
Fasting Glucose	96.50 (92.50–106.75)	100.00 (90.00–119.00)	0.480
HbA1c	5.50 (5.23–5.90)	5.90 (5.15–6.50)	0.620
HOMA-IR	2.10 (1.50–3.22)	3.10 (1.70–6.15)	0.140
Vitamin D	22.50 (19.00–30.75)	21.00 (16.50–25.50)	0.140

Abbreviations: age—participant age in years; BMI—body mass index; WHR—waist-to-hip ratio; TC—total cholesterol (mg/dL); HDL-C—high-density lipoprotein cholesterol (mg/dL); TC/HDL-C—total cholesterol to high-density lipoprotein ratio; SBP—systolic blood pressure (mmHg); QRISK—cardiovascular risk score (%); relative risk—relative cardiovascular risk score; non-HDL-C—non-high-density lipoprotein cholesterol (mg/dL); LDL-C—low-density lipoprotein cholesterol (mg/dL); triglycerides—serum triglycerides (mg/dL); uric acid—serum uric acid level (mg/dL); serum creatinine—serum creatinine level (mg/dL); fasting glucose—fasting blood glucose level (mg/dL); HbA1c—hemoglobin A1c (%); HOMA-IR—homeostatic model assessment of insulin resistance; vitamin D—serum vitamin D level (ng/mL); TRE—time-restricted eating; KD—ketogenic diet; WC—waist circumference.

**Table 2:** Baseline categorical characteristics of participants in the time-restricted eating and ketogenic diet groups.

Variable	Class	IF	KD	*p*-Value
Sex	F	17 (65.38%)	(69.57%)	0.760
	M	9 (34.62%)	7 (30.43%)	
Smoker	Yes	17 (65.38%)	13 (56.52%)	0.790
Sedentary	Yes	10 (38.46%)	8 (34.78%)	0.790
Sleep Deficit	Yes	12 (46.15%)	8 (34.78%)	0.420
Menopause	Yes	7 (26.92%)	5 (21.74%)	0.670
FMH CV	Yes	14 (53.85%)	7 (30.43%)	0.100
RA	Yes	8 (30.77%)	2 (8.7%)	0.060
LES	Yes	9 (34.62%)	6 (26.09%)	0.520
ED	Yes	5 (19.23%)	2 (8.7%)	0.290
Migraines	Yes	16 (61.54%)	3 (13.04%)	<0.001
AF	Yes	1 (3.85%)	0 (0%)	0.340

Abbreviations: sex—participant sex (F: female, M: male); smoker—smoking status (Yes: current smoker); sedentary—sedentary behavior (Yes: presence of a predominantly inactive lifestyle); sleep deficit—self-reported insufficient sleep (Yes: reported sleep deprivation); menopause—menopausal status (Yes: postmenopausal participant); FMH CV—family medical history of cardiovascular Disease (Yes: presence of a first-degree relative with cardiovascular disease); RA—rheumatoid arthritis (Yes: diagnosed with rheumatoid arthritis); LES—lupus erythematosus (Yes: diagnosed with systemic lupus erythematosus); ED—erectile dysfunction (Yes: diagnosed with erectile dysfunction); Migraines—presence of migraines (Yes: history of migraines); AF—atrial fibrillation (Yes: diagnosed with atrial fibrillation); KD—ketogenic diet.

**Table 3:** Longitudinal changes in study variables stratified by diet type.

Variable	Diet	% Change—Median (Q1,Q3)	Wilcoxon Signed Rank	Mann–Whitney U
BMI	TRE	4.06 (2.41, 6.49)	<0.001	<0.001
	KD	12.71 (9.84, 16.66)	<0.001	
WC	TRE	1.73 (0.91, 3.15)	<0.001	<0.001
	KD	11.43 (8.75, 13.45)	<0.001	
WHR	TRE	2.33 (0.28, 5.65)	<0.001	<0.001
	KD	11.22 (6.42, 19.21)	<0.001	
TC	TRE	2.04 (0.23, 3.99)	<0.001	<0.001
	KD	18.03 (14.89, 19.88)	<0.001	
HDL-C	TRE	−2.07 (−5.02, 1.62)	<0.001	<0.001
	KD	12.73 (8.33, 20.2)	<0.001	
TC/HDL-C	TRE	−0.76 (−2.78, 4.67)	<0.001	<0.001
	KD	25.74 (23.13, 30.5)	<0.001	
SBP	TRE	1.42 (0, 2.94)	<0.001	<0.001
	KD	8.97 (3.41, 12.5)	<0.001	
QRISK	TRE	2.16 (0.74, 8.03)	<0.001	<0.001
	KD	38.98 (29.79, 45.38)	<0.001	
Relative Risk	TRE	1.61 (0.58, 5.44)	<0.001	<0.001
	KD	26.76 (18.74, 44.78)	<0.001	
HbA1c	TRE	1.58 (0, 1.95)	<0.001	<0.001
	KD	8.33 (2.72, 13.6)	<0.001	

Abbreviations: BMI—body mass index; WC—waist circumference; WHR—waist-to-hip ratio; TC—total cholesterol (mg/dL); HDL-C—high-density lipoprotein cholesterol (mg/dL); TC/HDL-C—total cholesterol to high-density lipoprotein ratio; SBP—systolic blood pressure (mmHg); QRISK—cardiovascular risk score (%); relative risk—relative cardiovascular risk score; HbA1c—hemoglobin A1c (%); TRE—time-restricted eating; KD—ketogenic diet.

**Table 4:** Post hoc power analysis of Mann–Whitney U tests for longitudinal changes in study variables.

Variable	U Statistics	Mann–Whitney U	Rank-Biserial Correlation	Post Hoc Power
BMI Change	45.0	<0.001	0.849	0.804
WC Change	1.0	<0.001	0.997	0.911
WHR Change	106.5	<0.001	0.644	0.570
TC Change	6.0	<0.001	0.980	0.901
HDLc Change	22.0	<0.001	0.926	0.867
TC/HDLc Change	12.0	<0.001	0.960	0.889
SBP Change	85.0	<0.001	0.716	0.660
QRISK3 Change	48.5	<0.001	0.838	0.794
Relative Risk Change	29.0	<0.001	0.903	0.850
HbA1c Change	103.5	<0.001	0.654	0.584

Abbreviations: BMI—body mass index; WC—waist circumference; WHR—waist-to-hip ratio; TC—total cholesterol (mg/dL); SBP—systolic blood pressure (mmHg); QRISK—cardiovascular risk score (%); relative risk—relative cardiovascular risk score; HbA1c—hemoglobin A1c (%); Mann–Whitney U—non-parametric test comparing two independent groups; rank-biserial correlation—effect size measure for Mann–Whitney U test; post hoc power—statistical power of the Mann–Whitney U test; change—difference between post-intervention and baseline values (final value–initial value).

## Data Availability

The original contributions presented in this study are included in the article. Further inquiries can be directed to the corresponding author.

## Abbreviations

The following abbreviations are used in this manuscript: BMI—body mass index; WHR—waist-to-hip ratio; TC—total cholesterol (mg/dL); HDL-C—high-density lipoprotein cholesterol (mg/dL); TC/HDL-C—total cholesterol to high-density lipoprotein ratio; SBP—systolic blood pressure (mmHg); QRISK—cardiovascular risk score (%); relative risk—relative cardiovascular risk score; non-HDL-C—non-high-density lipoprotein cholesterol (mg/dL); LDL-C—low-density lipoprotein cholesterol (mg/dL); triglycerides—serum triglycerides (mg/dL); uric acid—serum uric acid level (mg/dL); serum creatinine—serum creatinine level (mg/dL); fasting glucose—fasting blood glucose level (mg/dL); HbA1c—hemoglobin A1c (%); HOMA-IR—homeostatic model assessment of insulin resistance; vitamin D—serum vitamin D level (ng/mL); TRE- time-restricted eating; KD—ketogenic diet; WC—waist circumference.

## References

[1] Kleinert S., Horton R. (2015). Obesity: A major global health challenge. Lancet.

[2] Pigeyre M., Yazdi F.T., Kaur Y., Meyre D. (2016). Recent progress in genetics, epigenetics and metagenomics unveils the pathophysiology of human obesity. Clin. Sci..

[3] Hubert H.B., Feinleib M., McNamara P.M., Castelli W.P. (1983). Obesity as an independent risk factor for cardiovascular disease: A 26-year follow-up of participants in the Framingham Heart Study. Circulation.

[4] Kusminski C.M., Perez-Tilve D., Müller T.D., DiMarchi R.D., Tschöp M.H., Scherer P.E. (2024). Transforming obesity: The advancement of multi-receptor drugs. Cell.

[5] Brüning J.C., Fenselau H. (2023). Integrative neurocircuits that control metabolism and food intake. Science.

[6] Busetto L., Dicker D., Frühbeck G., Halford J.C.G., Sbraccia P., Yumuk V., Goossens G.H. (2024). A new framework for the diagnosis, staging and management of obesity in adults. Nat. Med..

[7] Neufeld L.M., Hendriks S., Hugas M., Von Braun J., Afsana K., Fresco L.O., Hassan M.H.A. (2023). Healthy Diet: A Definition for the United Nations Food Systems Summit 2021. Science and Innovations for Food Systems Transformation [Internet].

[8] Bachar A., Birk R. (2025). Ketogenic Diet Intervention for Obesity Weight-Loss—A Narrative Review, Challenges, and Open Questions. Curr. Nutr. Rep..

[9] Westman E.C., Feinman R.D., Mavropoulos J.C., Vernon M.C., Volek J.S., Wortman J.A., Yancy W.S., Phinney S.D. (2007). Low-carbohydrate nutrition and metabolism. Am. J. Clin. Nutr..

[10] Masood W., Annamaraju P., Uppaluri K.R. (2023). Ketogenic Diet. StatPearls [Internet].

[11] Oh R., Gilani B., Uppaluri K.R. (2025). Low-Carbohydrate Diet. [Updated 17 August 2023]. StatPearls [Internet].

[12] O’Neill B., Raggi P. (2020). The ketogenic diet: Pros and cons. Atherosclerosis.

[13] Ezpeleta M., Cienfuegos S., Lin S., Pavlou V., Gabel K., Tussing-Humphreys L., Varady K.A. (2024). Time-restricted eating: Watching the clock to treat obesity. Cell Metab..

[14] Chaix A., Manoogian E.N.C., Melkani G.C., Panda S. (2019). Time-Restricted Eating to Prevent and Manage Chronic Metabolic Diseases. Annu. Rev. Nutr..

[15] Patikorn C., Roubal K., Veettil S.K., Chandran V., Pham T., Lee Y.Y., Giovannucci E.L., Varady K.A., Chaiyakunapruk N. (2021). Intermittent Fasting and Obesity-Related Health Outcomes: An Umbrella Review of Meta-analyses of Randomized Clinical Trials. JAMA Netw. Open.

[16] Varady K.A., Cienfuegos S., Ezpeleta M., Gabel K. (2022). Clinical application of intermittent fasting for weight loss: Progress and future directions. Nat. Rev. Endocrinol..

[17] Heymsfield S.B., Gonzalez M.C., Shen W., Redman L., Thomas D. (2014). Weight loss composition is one-fourth fat-free mass: A critical review and critique of this widely cited rule. Obes. Rev..

[18] Templeman I., Smith H.A., Chowdhury E., Chen Y.C., Carroll H., Johnson-Bonson D., Hengist A., Smith R., Creighton J., Clayton D. (2021). A randomized controlled trial to isolate the effects of fasting and energy restriction on weight loss and metabolic health in lean adults. Sci. Transl. Med..

[19] Olgin J.E., Wu N., Weiss E.J., Heymsfield S.B., Philip E., Lowe D.A., Moore A.H., Vittinghoff E., Shepherd J.A., Kelly N. (2020). Effects of Time-Restricted Eating on Weight Loss and Other Metabolic Parameters in Women and Men With Overweight and Obesity: The TREAT Randomized Clinical Trial. JAMA Intern. Med..

[20] Hippisley-Cox J., Coupland C., Brindle P. (2017). Development and validation of QRISK3 risk prediction algorithms to estimate future risk of cardiovascular disease: Prospective cohort study. BMJ.

[21] ClinRisk Ltd (2017). QRISK3-2018 Risk Calculator [Internet].

[22] Collins G.S., Altman D.G. (2009). An independent external validation and evaluation of QRISK cardiovascular risk prediction: A prospective open cohort study. BMJ.

[23] D’Agostino R.B., Vasan R.S., Pencina M.J., Wolf P.A., Cobain M., Massaro J.M., Kannel W.B. (2008). General cardiovascular risk profile for use in primary care: The Framingham Heart Study. Circulation.

[24] Estruch R., Ros E., Salas-Salvadó J., Covas M.I., Corella D., Arós F., Gómez-Gracia E., Ruiz-Gutiérrez V., Fiol M., Lapetra J. (2018). Primary Prevention of Cardiovascular Disease with a Mediterranean Diet Supplemented with Extra-Virgin Olive Oil or Nuts. N. Engl. J. Med..

[25] Holt R.I., Bushe C., Citrome L. (2005). Diabetes and schizophrenia 2005: Are we any closer to understanding the link?. J. Psychopharmacol..

[26] Mitchell S.L., Leon D.A.C., Chaugai S., Kawai V.K., Levinson R.T., Wei W.Q., Stein C.M. (2020). Pharmacogenetics of hypoglycemia associated with sulfonylurea therapy in usual clinical care. Pharmacogenomics J..

[27] Magiakou M.A., Mastorakos G., Oldfield E.H., Gomez M.T., Doppman J.L., Cutler G.B., Nieman L.K., Chrousos G.P. (1994). Cushing’s syndrome in children and adolescents. Presentation, diagnosis, and therapy. N. Engl. J. Med..

[28] (2024). Alcohol Use and Your Health. https://www.cdc.gov/alcohol/about-alcohol-use/index.html.

[29] Watson N.F., Badr M.S., Belenky G., Bliwise D.L., Buxton O.M., Buysse D., Dinges D.F., Gangwisch J., Grandner M.A., Kushida C. (2015). Recommended Amount of Sleep for a Healthy Adult: A Joint Consensus Statement of the American Academy of Sleep Medicine and Sleep Research Society. Sleep.

[30] Jagannathan K., Aydogan G., Spilka N., Kranzler H.R., Koellinger P.D., Nave G., Wetherill R.R., Daviet R. (2022). Associations between alcohol consumption and gray and white matter volumes in the UK Biobank. Nat. Commun..

[31] Kuriyan R. (2018). Body composition techniques. Indian J. Med. Res..

[32] Weir C.B., Jan A. (2025). BMI Classification Percentile And Cut Off Points. StatPearls [Internet].

[33] QRISK3 Calculator University of Nottingham. https://qrisk.org/.

[34] Andreotti F., Crea F., Patti G., Shoulders C.C., Navarese E.P., Robishaw J., Maseri A., Hennekens C.H. (2021). Family history in first degree relatives of patients with premature cardiovascular disease. Int. J. Cardiol..

[35] Ene C. (2014). Food Labeling in Romania in Relation to Consumer Protection. Econ. Insights Trends Chall..

[36] Gerrior S., Juan W., Basiotis P. (2006). An easy approach to calculating estimated energy requirements. Prev. Chronic Dis..

[37] (2005). Human energy requirements: Report of a joint FAO/WHO/UNU Expert Consultation. Food Nutr. Bull..

[38] Mozaffarian D., Micha R., Wallace S. (2010). Effects on coronary heart disease of increasing polyunsaturated fat in place of saturated fat: A systematic review and meta-analysis of randomized controlled trials. PLoS Med..

[39] FAO (2013). Dietary protein quality evaluation in human nutrition: Report of an FAO Expert Consultation. FAO Food Nutr Pap..

[40] Powell-Wiley T.M., Poirier P., Burke L.E., Després J.P., Gordon-Larsen P., Lavie C.J., Lear S.A., Ndumele C.E., Neeland I.J., Sanders P. (2021). Obesity and Cardiovascular Disease: A Scientific Statement from the American Heart Association. Circulation.

[41] Hall J.E., do Carmo J.M., da Silva A.A., Wang Z., Hall M.E. (2015). Obesity-induced hypertension: Interaction of neurohumoral and renal mechanisms. Circ. Res..

[42] Mu X., Wu A., Hu H., Zhou H., Yang M. (2022). Assessment of QRISK3 as a predictor of cardiovascular disease events in type 2 diabetes mellitus. Front. Endocrinol..

[43] Sadeghi M., Sarrafzadegan N., Mohebian M.R., OveisGharan S., Mansourian M., Talaei M., Masoudkabir F., Hassannejad R., Marateb H.R., Roohafza H.R. (2017). PARS risk charts: A 10-year study of risk assessment for cardiovascular diseases in Eastern Mediterranean Region. PLoS ONE.

[44] Kasim S.S., Ibrahim N., Malek S., Ibrahim K.S., Aziz M.F., Song C., Chia Y.C., Ramli A.S., Negishi K., Mat Nasir N. (2023). Validation of the general Framingham Risk Score (FRS), SCORE2, revised PCE and WHO CVD risk scores in an Asian population. Lancet Reg. Health West. Pac..

[45] Stefan N., Häring H.U., Hu F.B., Schulze M.B. (2013). Metabolically healthy obesity: Epidemiology, mechanisms, and clinical implications. Lancet Diabetes Endocrinol..

[46] Wu G., Wu J., Lu Q., Cheng Y., Mei W. (2023). Association between cardiovascular risk factors and atrial fibrillation. Front. Cardiovasc. Med..

[47] Parsons R.E., Liu X., Collister J.A., Clifton D.A., Cairns B.J., Clifton L. (2023). Independent external validation of the QRISK3 cardiovascular disease risk prediction model using UK Biobank. Heart.

[48] Zinman B., Wanner C., Lachin J.M., Fitchett D., Bluhmki E., Hantel S., Mattheus M., Devins T., Johansen O.E., Woerle H.J. (2015). Empagliflozin, Cardiovascular Outcomes, and Mortality in Type 2 Diabetes. N. Engl. J. Med..

[49] O’Donovan G., Lee I.M., Hamer M., Stamatakis E. (2017). Association of “Weekend Warrior” and Other Leisure Time Physical Activity Patterns with Risks for All-Cause, Cardiovascular Disease, and Cancer Mortality. JAMA Intern Med..

[50] Woodward M., Brindle P., Tunstall-Pedoe H., SIGN Group on Risk Estimation (2007). Adding social deprivation and family history to cardiovascular risk assessment: The ASSIGN score from the Scottish Heart Health Extended Cohort (SHHEC). Heart.

[51] Kim J.Y. (2021). Optimal Diet Strategies for Weight Loss and Weight Loss Maintenance. J. Obes. Metab. Syndr..

[52] Makris A., Foster G.D. (2011). Dietary approaches to the treatment of obesity. Psychiatr. Clin. N. Am..

[53] Volek J.S., Vanheest J.L., Forsythe C.E. (2005). Diet and exercise for weight loss: A review of current issues. Sports Med..

[54] Wirth A., Wabitsch M., Hauner H. (2014). The prevention and treatment of obesity. Dtsch. Arztebl. Int..

[55] Wang Z., Chen T., Wu S., Dong X., Zhang M., Ma G. (2024). Impact of the ketogenic diet as a dietary approach on cardiovascular disease risk factors: A meta-analysis of randomized clinical trials. Am. J. Clin. Nutr..

[56] Gabel K., Cienfuegos S., Kalam F., Ezpeleta M., Varady K.A. (2021). Time-Restricted Eating to Improve Cardiovascular Health. Curr. Atheroscler. Rep..

